# Parameter Optimization for Quantitative Signal-Concentration Mapping Using Spoiled Gradient Echo MRI

**DOI:** 10.1155/2012/815729

**Published:** 2012-11-05

**Authors:** Gasser Hathout, Neema Jamshidi

**Affiliations:** UCLA Department of Radiology, UCLA Center for Health Sciences, 10833 Le Conte Avenue, Los Angeles, CA 90095, USA

## Abstract

*Rationale and Objectives*. Accurate signal to tracer concentration maps are critical to quantitative MRI. The purpose of this study was to evaluate and optimize spoiled gradient echo (SPGR) MR sequences for the use of gadolinium (Gd-DTPA) as a kinetic tracer. *Methods*. Water-gadolinium phantoms were constructed for a physiologic range of gadolinium concentrations. Observed and calculated SPGR signal to concentration curves were generated. Using a percentage error determination, optimal pulse parameters for signal to concentration mapping were obtained. *Results*. The accuracy of the SPGR equation is a function of the chosen MR pulse parameters, particularly the time to repetition (TR) and the flip angle (FA). At all experimental values of TR, increasing FA decreases the ratio between observed and calculated signals. Conversely, for a constant FA, increasing TR increases this ratio. Using optimized pulse parameter sets, it is possible to achieve excellent accuracy (approximately 5%) over a physiologic range of concentration tracer concentrations. *Conclusion*. Optimal pulse parameter sets exist and their use is essential for deriving accurate signal to concentration curves in quantitative MRI.

## 1. Introduction

In the recent years, MR has transitioned from an anatomic imaging modality to one also capable of performing advanced functional applications. Among these advanced applications are perfusion imaging, which has become a significant tool in the assessment of both stroke and tumor patients, and permeability imaging, used in a wide array of applications. In these applications, gadolinium diethylenetriamine pentaacetic acid (Gd-DTPA) is typically injected intravenously and used as a kinetic tracer, with serial images obtained to track the washin and washout of gadolinium from the region of interest. The mathematical tools of flow modeling and compartmental modeling are then used to provide relevant parameters for assessment of cerebral blood flow, cerebral blood volume, and vascular permeability. 

Central to reliable kinetic modeling is the establishment of an accurate method of converting the observed signal changes from the passage of the gadolinium bolus into an accurate tracer concentration versus time curve. Thus far, this issue has not received sufficient attention in the literature. In previous studies, the signal-concentration curve has either been derived invasively, by direct blood, urine, or tissue measurements using radioactive gadolinium-153 [1], or by correlation with a standard curve obtained by serial dilutions [2, 3], or been calculated noninvasively, relying on equations characterizing the relaxivities of gadolinium (*R*1 and *R*2) and the pulse sequence used in the particular study [4–8].

This paper seeks to further refine the mathematical calculation of signal-concentration curves for Gd-DTPA using spoiled gradient recalled echo (SPGR) pulse sequences by systematically studying the questions surrounding pulse-sequence optimization for quantitative signal-concentration mapping.

One central concern is that there may be a tacit assumption that the MR signal equations used to convert tissue signal to gadolinium concentration are equally valid over a large range of sequence parameters [5, 8], allowing a convenient and reasonable set of parameters to be employed for calculation, without rigorous testing as to whether identical results would be obtained for other imaging parameter sets. Furthermore, such an assumption is made despite the fact that the gradients applied during imaging often have significant differences from the idealized geometry assumed when using equations to predict observed tissue signal. 

Our paper seeks to analyze the capacity of the traditional SPGR signal equation for obtaining an accurate signal to concentration conversion under idealized circumstances where the gadolinium concentrations are accurately known and where we do not have to be concerned about such complicating factors as flow artifacts, compartmental exchange rates, or recirculation and second pass effects. Our assumption is that if accurate signal-concentration maps cannot be obtained under even idealized circumstances, then quantitative MR assessment of perfusion and permeability parameters may not be possible.

We used a simple Gd-DTPA in H_2_O phantom to address a number of questions. Primarily, is the SPGR signal equation universally valid across imaging parameter sets, or is there an optimal set of pulse sequence parameters for generating a signal-concentration map? If an optimal set of parameters exists, can this be determined theoretically or must it be determined empirically? Moreover, we seek to understand what is the accuracy of such an “optimal parameter set” over a range of physiologically relevant tracer concentrations, and to estimate how gross is the error in calculated tracer concentrations when suboptimal pulse parameters are chosen. This aggregate of questions allows us to assess whether it is possible to produce an accurate signal to concentration map using MRI under idealized conditions.

## 2. Materials and Methods

To accurately answer the questions posed above, we wanted to calculate all of the relevant parameters de novo and to avoid relying on published estimates of either *T*1 or *T*2 relaxation times for water-gadolinium mixtures or published relaxivities for gadolinium. The aim was to be sure that any deviations between predicted and observed MR signal were not due to differences between such published values and what would have been calculated on our MR platform. Therefore, our experimental plan is first described in general terms, with the details of each step subsequently provided:

1. We prepared carefully a doped Gd-DTPA water phantom with multiple water vials with known graded concentrations of gadolinium.
2. An inversion recovery (IR) set of pulse sequences with variable times to inversion was used to calculate the *T*1 times of the tubes in the phantom and to produce a *T*1 versus gadolinium concentration curve. Exponential curve fitting was used to calculate *R*1, the gadolinium relaxivity of gadolinium related to *T*1.
3. The phantom was then imaged with a spin echo-variable echo (SE/VE) pulse sequence with variable times to echo (TE) to calculate the *T*2 times for each tube in the phantom and to produce a *T*2 versus gadolinium concentration curve. Using the previously calculated values of *T*1 and *R*1, the *T*2-associated relaxivity of gadolinium, *R*2, was then calculated.
4. Multiple SPGR sequences were run on the phantom, with varying pulse parameters: variable times to repetition (TR), TE, and flip angles. Signal versus concentration curves were produced for each SPGR pulse sequence. These observed signal-concentration curves are labeled as SIGOBS.
5. Using our calculated values of *R*1 and *R*2, as well as the known pulse sequence parameters (TR, TE, and flip angle), the SPGR signal equation was used to produce a calculated MR signal-concentration curve. This is called SIG. This is then compared to the observed signal to assess the accuracy of the SPGR signal equation in obtaining an MR signal-concentration curve.
6. Finally, a more robust form of the gradient echo signal equation is assessed to see whether the results would deviate significantly from the more compact SPGR signal equation.

### 2.1. Phantom Preparation

A Gd-DTPA phantom was prepared using plain test tubes microtitrated to 7 mL H_2_O doped with contrast, Gd-DTPA (Magnevist Berlex Imaging, Wayne, NJ, USA). The concentration range prepared was 0–8 mM/L, with greater sampling of the physiologic range 0–2 mM/L. 

#### 2.1.1. Imaging

All imaging was performed on a conventional GE Signa 1.5T MRI scanner (Milwaukee, WI), using an extremity coil. All pulse sequences were performed twice, and the results averaged. The following pulse sequences were used to image each phantom.

- IR sequence—TR 3000 ms, TE 18, TI 2000, 1000, 850, 700, 500, 300, 100, 50 ms. The TE was kept short in order to minimize the dependence of the sequence on T2 signal.
- SE/VE sequences—TR 3000 ms, TE 20, 25, 40, 50, 60, 75, 100 ms.
- SPGR sequences—176 imaging sequences were performed over a range of TR, TE, and flip angles (FA), ranging from TR 40–200 ms, TE 5–30 ms, and FA 10°–90°. Sample pulse sequences include:
- TR 40 ms, TE 10 ms, FA: 10°, 20°, 30°, 40°, 50°, 60°, and 90°;
- TR 100 ms, TE 10 ms, FA: 10°, 20°, 30°, 40°, 50°, 60°, and 90°.

### 2.2. *T*1 and *R*1 Determination

IR sequence imaging with variable times to inversion was used to determine *T*1 times for each Gd-DTPA concentration in our phantom set, generating a *T*1-concentration curve. Fitting for *T*1 was done using the IR equation and using the two parameter least squares fitting routine:

(1) $$\begin{array}{c} S=S_{0}\left(1-2e^{-\text{TI}/T1}+e^{-\text{TR}/T1}\right). \end{array}$$

Since the scanner does not distinguish between positive and negative signals, each dataset was plotted and manually analyzed, and the negative values were adjusted by inverting the appropriate values with respect to the null point. 

*R*1 was determined from the mean *T*1-concentration curve by fitting the following equation:

(2) $$\begin{array}{c} \frac{1}{T1\left(i\right)}=\frac{1}{T1_{0}}+R1*C\left(i\right), \end{array}$$

where *T*1(*i*) is the *T*1 time at a given gadolinium concentration, [Gd-DTPA] = *C*(*i*), and *i* is the *i*th concentration value. 

### 2.3. *T*2 and *R*2 Determination

SE/VE sequence imaging with variable TEs was used to determine *T*2 times using the previously determined *T*1's and the SE equation with a two-parameter least squares fit:

(3) $$\begin{array}{c} S=S_{0}\;e^{-\text{TE}/T2}\;\left(1-e^{-\text{TR}/T1}\right). \end{array}$$

*R*2 was determined from the *T*2-concentration curve by fitting the following equation:

(4) $$\begin{array}{c} \frac{1}{T2\left(i\right)}=\frac{1}{T2_{0}}+R2*C\left(i\right), \end{array}$$

where the *T*2(*i*) is the *T*2 time at each gadolinium concentration [Gd-DTPA] = *C*(*i*).

### 2.4. Analysis of SPGR Data

SPGR signal data was obtained using multiple sequence parameters (TR, TE, FA) over the range of Gd-DPTA concentrations *C*(*i*) = 0–8 mM/L. This observed signal is designated SIGOBS(*i*). SIGOBS(0) represents signal at zero tracer concentration. 

The predicted or theoretical SPGR signal for each Gd-DPTA concentration, designated SIG(*i*), was calculated from the following SPGR signal equation, as well as the calculated initial values of *T*1 and *T*2 (*T*1_0_, *T*2_0_), and the calculated *R*1 and *R*2 for the Gd-DTPA phantom [9]:

(5) $$\begin{array}{c} \text{SIG}\left(i\right)=\frac{S_{0}\left(\sin\alpha \right)\;\left(1-e^{-(\text{TR}/T1(i))}\right)\left(e^{-(\text{TE}/T2(i))}\right)}{1-\cos\alpha \left(e^{-(\text{TR}/T1(i))}\right)}, \end{array}$$

where it is assumed that *α* is the flip angle,

*T*2* is approximated by *T*2,
*T*1 (*i*) = 1/(1/*T*1_0_ + *R*1∗*C*(*i*)),
*T*2 (*i*) = 1/(1/*T*20 + *R*2∗*C*(*i*)),

and where *S*_0_ is calculated to normalize SIG(0) to SIGOBS(0). 

### 2.5. Parameter Optimization

For each of the SPGR pulse sequences examined, comparison was made between the observed signal, SIGBOBS(*i*), and the calculated signal, SIG(*i*), at the relevant pulse sequence parameters. 

Then, for each pulse sequence, a percentage error determination (PD) was calculated between SIGOBS and SIG, both for each concentration and as an average absolute error over the entire concentration range:

(6) $$\begin{array}{c} \text{PD}\left(i\right)=\frac{\text{SIGOBS}\left(i\right)-\text{SIG}\left(i\right)}{\text{SIGOBS}\left(i\right)}. \end{array}$$

### 2.6. Analytical versus Empirical Optimization

The effect of TR, TE, and FA on the concordance of the observed (SIGOBS) and predicted (SIG) signals was evaluated. Optimal pulse parameter sets were identified as those yielding the lowest average absolute error over the entire concentration range. 

To determine whether these pulse parameter sets could be theoretically derived, the parameters maximizing overall signal in the SPGR equation were calculated and compared to the empirically determined optimal pulse parameter sets. 

Finally, in an effort to improve the results of analytical optimization and to better understand the systematic variations in pulse sequence accuracy as a function of TR and FA, a more general form of the gradient echo equation is investigated to see if it would improve the fits obtained by the optimal pulse sequences [9]:

(7) $$\begin{array}{c} S=\int_{0}^{2\pi }\frac{S_{0}\left(\sin\alpha \right)\;\left(1-e^{-(\text{TR}/T1(i))}\right)\left(e^{-(\text{TE}/T2(i))}\right)}{1-\cos\alpha \left(e^{-(\text{TR}/T1(i))}\right)-W\left(\theta \right)}g\left(\theta \right)d\theta , \end{array}$$

where

(8) $$\begin{array}{c} W\left(\theta \right)=\frac{\left(e^{-(\text{TE}/T2(i))}\right)\;\left(e^{-(\text{TR}/T1(i))}-\cos\alpha \right)\left(e^{-(\text{TE}/T2(i))}-\cos\theta \right)}{1-\cos\theta \left(e^{-(\text{TE}/T2(i))}\right)}. \end{array}$$

Here, *α* is the FA, and *θ* is the angle of precession in the *x*-*y* plane between pulses and accounts for all such precession, whether it arises from off-resonance effects due to field inhomogeneities and susceptibility variations or from any field gradients applied between pulses [9]. 

Using (7) directly is untenable due to the inability to define *g*(*θ*)*d*(*θ*), which represents the fraction of spins with precession angle between *θ* and *θ* + *dθ*. If, however, one deals with spoiled gradient echo imaging, the spoiler pulse disperses transverse magnetization after data collection, producing a state where all precession angles are equally likely and *g*(*θ*) = 1/(2*π*); hence, the resulting signal equation can be expressed as [9]

(9) $$\begin{array}{c} S=\int_{0}^{2\pi }\frac{1}{2\pi }\frac{S_{0}\left(\sin\alpha \right)\;\left(1-e^{-(\text{TR}/T1(i))}\right)\left(e^{-(\text{TE}/T2(i))}\right)}{1-\cos\alpha \left(e^{-(\text{TR}/T1(i))}\right)-W\left(\theta \right)}d\theta , \end{array}$$

where once again, the effects of inherent inhomogeneity within each voxel not accounted for by the above equation are assumed to be negligible. 

## 3. Results

### 3.1. Relaxation Times and Relaxivity Estimates

**Figure 1:**
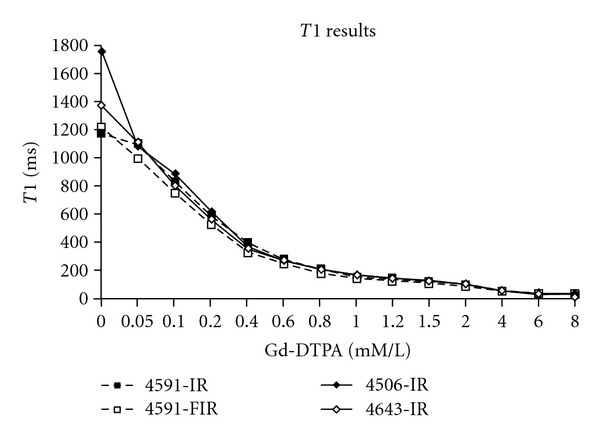
Calculated *T*1 values versus Gd-DTPA concentration for several experimental runs, labeled by the run number in our original database. The individual runs, rather than the averaged values, are shown to stress that the curves follow the same pattern of decreasing *T*1 with increasing gadolinium concentrations in a fairly smooth form. Values of *T*1 are obtained from a least squares fit of (1) (Section 2).

**Figure 2:**
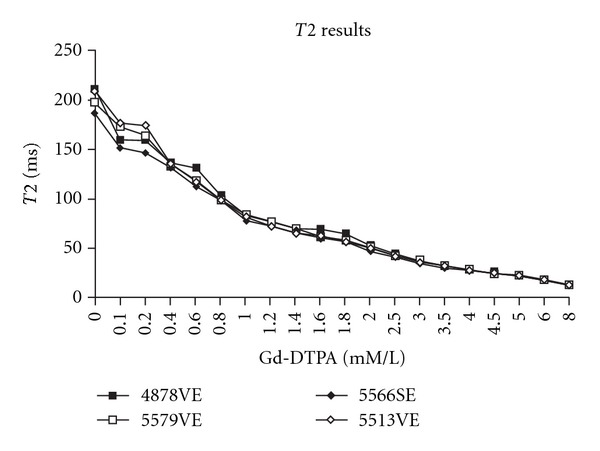
Calculated *T*2 values versus Gd-DTPA concentration for several experimental runs. The individual runs, rather than the averaged values, are shown to stress that the curves follow the same pattern of decreasing *T*2 with increasing gadolinium concentrations in a fairly smooth form. Values of *T*2 are obtained from a least squares fit of (3) (Section 2).

As noted in Section 2, IR imaging was used to determine *T*1 values for each concentration of Gd-DTPA, with *T*1 determined using the least squares fit for (1). The results are noted in Figure 1. As expected, there is a shortening of *T*1 with increasing gadolinium concentration. Similarly, using the calculated values of *T*1 and (3), *T*2 values for each Gd-DTPA concentration are calculated. The results are noted in Figure 2.

Using the above results as well as (2) and (4), *R*1 and *R*2 are calculated by least-squares fitting. The results are displayed in Table 1 along with reference values for comparison from Su et al. [10].

### 3.2. Parameter Optimization

**Figure 3:**
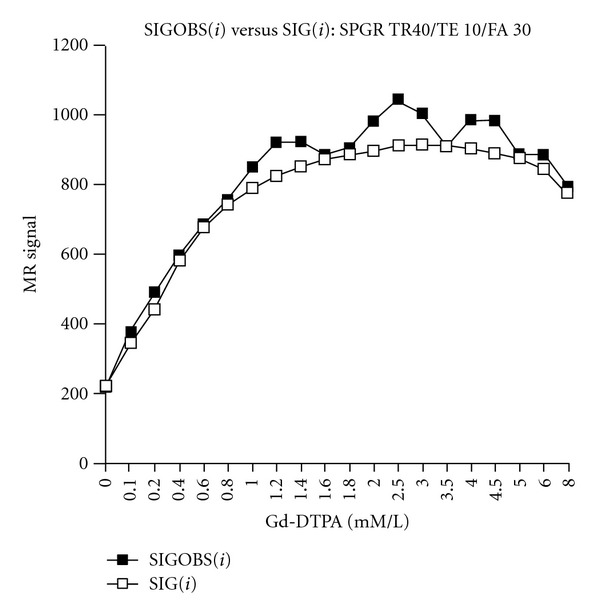
Observed (SIGOBS) versus calculated (SIG) signal for a sample experimental run (SPGR TR 40 ms/TE 10 ms/FA 30°). Using this optimal pulse sequence, there is excellent concordance between observed and predicted signal. This suggests that with optimal pulse sequences, it is possible to derive an accurate signal to concentration map, at least under idealized conditions.

**Figure 4:**
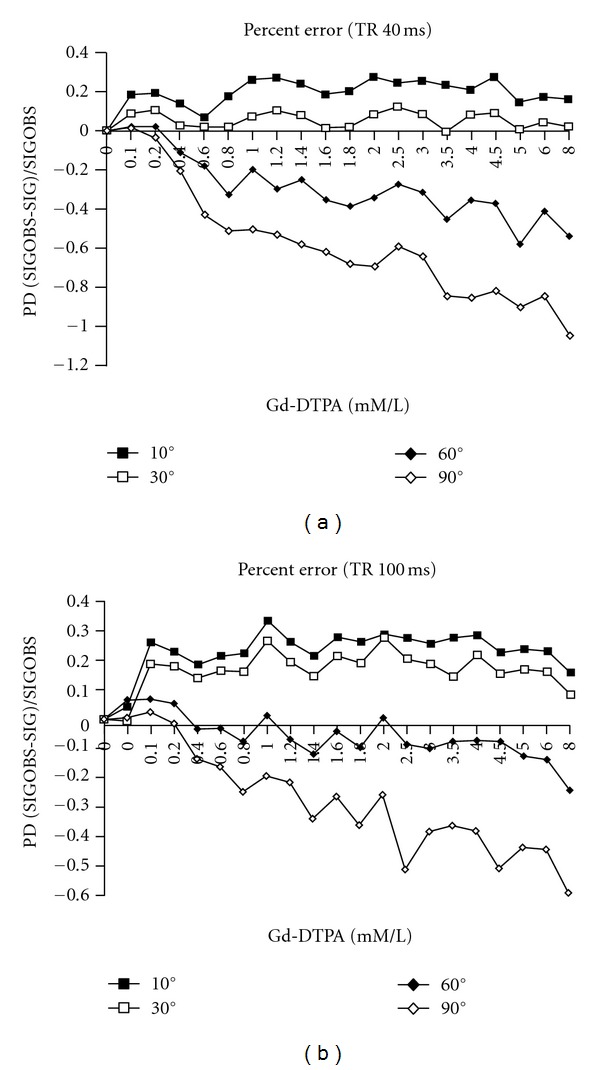
(a) Percentage difference between observed and calculated signals versus gadolinium concentration for a sample experimental run (SPGR TR 40 ms/TE 10 ms) for varying values of FA: 10°, 30°, 60°, and 90°. There is a systematic variation in percentage difference (PD) with increasing flip angle, with progressively decreasing ratios of observed to predicted signal. For a TR of 40 ms, the accuracy-optimal FA is 30°. At this flip angle, observed signal is only slightly greater than predicted signal. (b) Percentage difference between observed and calculated signals versus gadolinium concentration for another sample experimental run (SPGR TR 100 ms/TE 10 ms) for varying values of FA: 10°, 30°, 60°, and 90°. The same pattern of variation is seen as in (a), except that for a TR of 100 ms, the accuracy-optimal FA is 60°.

For each SPGR pulse sequence, (5) is used to calculate predicted signal (SIG), based on the values of the *T*1_0_, *T*2_0_, *R*1, and *R*2 obtained above. Predicted signal (SIG) is then compared to observed signal (SIGOBS) at each Gd-DTPA concentration (Figure 3 shows an example using an SPGR pulse sequence with TR 40 ms/TE 10 ms/FA 30°). Using (6), the percentage difference (PD) between predicted signal (SIG) and measured signals (SIGOBSs) is calculated for each SPGR sequence used. This allows an analysis of variation of percentage error with TR and FA. Figures 4(a) and 4(b) demonstrate the variation of PD with FA at TR values of 40 and 100 ms, respectively, for representative imaging runs.

These results demonstrate that there are systematic variations in PD between observed and predicted signals over the physiologic concentration of Gd-DTPA. With a TR of 40 ms, the most accurate fit is observed for an FA of 30°. The remaining sets yield significantly poorer fits. As the flip angle increases, the ratio of observed to calculated signal (SIGOBS to SIG) decreases. At an FA of 10° observed signal is uniformly greater than calculated signal. At an FA of 60° and 90°, the observed signal is significantly less than the calculated. At a TR of 100 ms, the same systematic variation of PD with FA is observed, but the most accurate fit is obtained with an FA of 60°. For a TR of 40 ms and 100 ms, Table 2 displays average absolute percentage error as a function of FA, averaged over all datasets. 

The demonstrated systematic relationship holds up at even finer increments of FA, as illustrated in Table 3, which represent the percentage error for an SPGR sequence of TR 70 ms, TE 10 ms, and FA of 10°, 20°, 30°, 40°, 50°, 60°, 70°, and 90°. The greatest accuracy is obtained when FA equals 50°, where PD is reduced to 5%.

### 3.3. Analytical versus Empirical Optimization

Given that there appear to be optimal pulse parameter sets that give lower percentage errors between observed and calculated signals, the question was posed as to whether these optimal pulse parameters were the ones which maximized signal in the SPGR signal equation.

**Figure 5:**
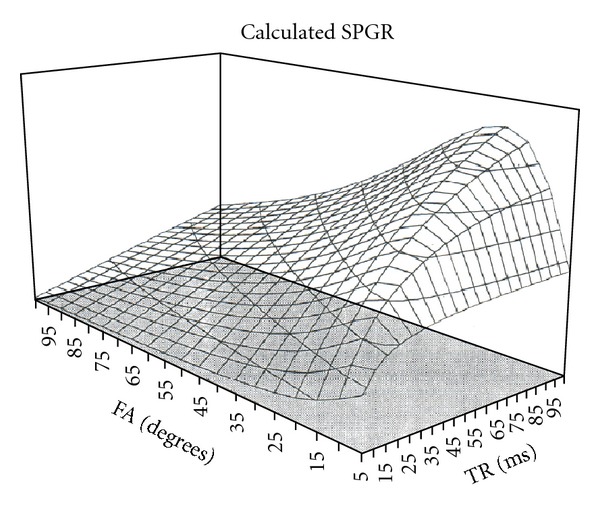
A manifold showing calculated SPGR signal using (5) as a function of TR and FA. For each value of TR, there is a corresponding FA which maximizes expected signal. It turns out that the sequence parameters which maximize SPGR signal are not the ones that give the most accurate signal-concentration curves (see Section 4).

Using (5), the parameters maximizing overall signal in the SPGR equation were calculated and compared to the optimal pulse parameter sets (Figure 5). It is noted that the optimal pulse parameter sets are not those which maximize signal (see Section 4). 

To further investigate possible theoretical reasons for the systematic variations in accuracy of the predicted signal-concentration curves with TR and FA, a more general form of the SPGR signal (9) was examined and compared to the results of (5). 

## 4. Discussion

While Gd-DTPA-enhanced MR imaging is routinely performed to answer the binary question of whether there is enhancement, dynamic MR imaging seeks to capture the additional information found in the kinetic properties of signal variation. This approach has been applied to various organ systems and at various levels of quantitative rigor, to aid in characterizing physiological and pathological states. Early studies of the dynamics of contrast enhancement sought to characterize renal pathology [11–20], assess myocardial ischemia [1, 19], and study the signal-time courses of CNS tumors in an attempt to enhance diagnostic accuracy [20, 21]. By and large, however, these studies were qualitative in nature, attempting to study the differences between dynamic signal time curves for disease characterization. 

Quantitative dynamic MR imaging, employing pharmacokinetic modeling with gadolinium-based contrast agents as kinetic tracers, has proven to be a more challenging undertaking. The construction of rigorous models requires an accurate signal-concentration curve for the kinetic tracer at hand, as well as information regarding compartmentalization of the gadolinium-based treatment tracer, the intercompartmental exchange rates, and the H_2_O exchange rates between the various compartments [19]. 

To date much excellent work has been directed at meeting these challenges, again in a variety of physiological applications, including estimates of renal function [2] and myocardial perfusion [19, 22]. Central to these applications is having an accurate MRI signal to tracer concentration conversion. In some papers, this has been done by comparing the MR signal to a known ex vivo standard dilution curve that relates MR signal to tracer concentration. For example, functional estimates of glomerular filtration rates have been performed using plasma and urine Gd-DTPA measurements related to a signal-concentration curve obtained by serial dilution [2]. Also, true quantitative estimates of myocardial blood flow have been obtained with pharmacokinetic models and signal-concentration curves obtained either by direct measurement using Gd-DTPA doped with radioactive 153-Gd-DTPA [1] or using standard reference preparation to generate a calibrated signal-concentration curve [3]. 

However, using exogenous reference preparations is cumbersome and time-consuming. An alternative approach to quantitative analysis is to use mathematical modeling to convert the observed signal-time curve into a gadolinium concentration-time curve. In this vein, the seminal work of Belliveau et al. and Rosen et al. [4, 7] established susceptibility-based quantitative imaging and generated relative CBV maps for the brain. Likewise, there have been excellent quantitative analyses of blood-brain barrier permeability in the CNS [5, 6, 8, 23]. Many of the early quantitative studies using calculated signal-concentration curves relied largely on inversion-recovery or spinecho sequences and on approximations using the spin-echo signal equation to derive a nearly linear relationship between signal and gadolinium concentration [5, 6]. Moreover, often the tacit assumption has been made that the sequence equations are equally valid over a large range of sequence parameters [5, 8], allowing a convenient and reasonable set of parameters to be employed for calculation, without rigorous testing as to whether identical results would be obtained for other imaging parameter sets. Possible drawbacks with such an approach include the possibility that in blood and tissue, the linearity assumption, although mathematically reasonable, may not be sufficiently accurate and that experimental validation is required [5]. 

For example, Schabel and Parker demonstrated that optimizing the flip angle can lead to improved SNR in DCE-MRI, thus demonstrating that there is some dependence of signal on the pulse parameters chosen [24]. Also, the work of Su et al. [10] suggests that the signal-concentration relationship is nonlinear and that the enhancement ratio is most accurately related to concentration by a log-log plot, which shows good linearity. Additionally, Su et al. tested the accuracy of a theoretically calculated signal-concentration curve using a spin-echo sequence (SE 100/18) and measured *R*1 and *R*2 rates in a phantom, against direct signal measurement in gel-bead phantoms of known gadolinium concentration. These authors noted some inaccuracy, with the theoretically calculated enhancement ratio being somewhat overestimated [10]. 

The situation with gradient echo imaging is at once more compelling. Flow measurements require imaging sequences fast enough to allow bolus tracking during the first pass of a contrast agent; otherwise the problem becomes significantly more difficult due to recirculation effects [1, 19]. Thus, if an accurate signal-concentration map was constructed, gradient-echo imaging (with its high acquisition speed) would be extremely useful in quantitative applications. However, for gradient echo sequences, the relationship between MR signal and Gd-DTPA is given by nonlinear function [5, 19], further complicating quantitative analysis.

Thus, the purpose of this study was to evaluate and optimize spoiled gradient-echo pulse sequences for the use of gadolinium as a kinetic tracer, specifically, to assess the accuracy of signal-concentration maps calculated analytically from the SPGR signal equation and the dependence of the accuracy of these maps on pulse parameters. Spoiled sequences were chosen for this initial analysis due to the fact that spoiling allows for simplifications in the form of the sequence equation and eliminates the need (at least in theory) to deal with residual transverse magnetization [9] (see Section 2.6).

Initially, we wished to examine the signal-concentration curve for the simplest mono-compartmental model available, Gd-DTPA diluted in test tubes of H_2_O, to provide water molecules with ready access to the coordination sites of the paramagnetic gadolinium atom in time frames which are relatively short compared with the observed relaxation times [7]. It is in these *T*1 and *T*2 “fast-exchange” regimes that a theoretical analysis using signal equations would be most accurate.

The basic methodology relied on calculating *T*1 and *T*2 times for each Gd-DTPA concentration in the phantom, to allow calculation of relaxivities *R*1 and *R*2 [1, 6, 8, 10, 25]. This allows the use of the relaxivity equations ((2) and (4)) to estimate changes in *T*1 and *T*2 with Gd-DTPA and to calculate predicted signal using an appropriate pulse sequence equation (e.g., (5) for SPGR).

If a signal-concentration curve can be verified as accurate using such theoretical methods, then once *T*1_0_, *T*2_0_, *R*1, and *R*2 are known, Gd-DTPA concentration can be accurately predicted from observed signal. As mentioned above, other significant difficulties, including issues of compartmentalization and the accurate determination of tissue *R*1 and *R*2, must be addressed before accurate *in vivo* work is possible. A critical initial step, however, is the verification of this basic method *in vitro*, as our work attempts.

Central to this approach is the assumption that *T*1 and *T*2 rate changes are linearly related to Gd-DTPA concentration, in other words that (2) and (4) are accurate. To date, multiple studies have verified the accuracy of this relationship, both in aqueous media, as well as strongly suggesting that the relationship is likewise valid for blood and tissue if a fast-exchange regime is present [5, 7, 26, 27].

Our study, likewise, verifies this relationship (Figures 1 and 2, and Table 1), with an estimated *R*1 of 0.0055/ms-mM, and an *R*2 of 0.0069/ms-mM. These values are in excellent agreement with those of the other investigative, such as Su et al. [10], who obtained *R*1 and *R*2 values of 0.0053/ms-mM and 0.0068/ms-mM, respectively, as well as in good agreement with the values in a broad range of other studies [1, 25].

However, our results clearly demonstrate that even when *R*1 and *R*2 are well known and the rate changes of *T*1 and *T*2 with Gd-DTPA concentration are well characterized, the SPGR signal equation is not universally valid, even across our limited range of parameter choices. Moreover, the results show that there exist optimal pulse sequence parameters for generating an accurate signal-concentration curve. Figures 4(a) and 4(b), as well as Table 2, demonstrate that there is a systematic variation of percentage error between observed and calculated signals as a function of FA for constant TR and TE. For example, at a TR 40 ms/TE 10 ms, the most accurate fit between predicted and observed signals occurs with an FA of 30°, while at TR 100 ms/TE 10 ms, the most accurate fit is obtained with an FA of 60°. Furthermore the values of PD vary quite systematically with TR and FA, even at fine incremental changes of FA (see Table 3). Thus, the accuracy of the SPGR equation is a function of TR and FA. 

Analysis of this data reveals that, but while both observed and predicted single curves change with variations in TR and FA, SIGOBS (observed signal) is more sensitive to parameter changes than SIG (predicted or calculated signal). Hence, the SPGR signal equation does not fully characterize the observed signal, and underestimates the impact of parameter changes on the observed signal. Thus at TR 40/FA 10 (Figure 4(a)), observed signal, is significantly greater than predicted single over the entire concentration range. Increasing FA to 30° decreases both observed and predicted signals, with a greater impact on observed than predicted signal. This produces a closer concordance between observed and predicted signal-concentration curves and yields an “optimal” pulse parameter set, with an average absolute error of only 5% over the entire concentration range and an excellent concordance at the lower (more physiologic) concentrations of 0–2 mM/L (Figure 3). Further increases in FA, for example, to 60° or 90°, continue to produce somewhat greater decreases in observed than predicted signal-concentration curves, such that the observed signal (SIGOBS) now becomes significantly less than the predicted signal over the entire concentration range (Figure 4(a)). Precisely analogous conclusions are drawn for pulse sequences employing a TR of 70 ms and TR of 100 ms (Table 3, and Figure 4(b) and Table 2, resp.), with the exception that for a TR of 70 ms, the optimal FA 50°, while for a TR of 100 ms, the optimal FA is 60°.

At all experimental values of TR, increasing FA tends to decrease the ratio between observed and calculated signals. Conversely, for a constant FA, increasing TR tends to increase the ratio of observed to calculated signal. Hence, in pulse sequence optimization, as chosen TR increases, the FA must likewise increase in order to maintain accuracy. Thus, for a TR of 40 ms, the optimal FA is 30°, for a TR of 70 ms, it is 50°, while for a TR of 100 ms, the optimal FA is 60°, as mentioned above. 

When using optimal pulse parameter sets, we find that there is excellent concordance between the observed and predicted signals, with average absolute error rates of 5–10%. Conversely, the use of suboptimal pulse parameters exacts a high price in terms of accuracy with the absolute error rates of 30–40% (Figures 3 and 4, Tables 2 and 3).

The effect of TE on the accuracy of SIG (predicted signal-concentration curve) was more difficult to characterize. TE values of 5 ms to 30 ms were studied at TRs of 40 ms and 100 ms. There was no true systematic variation, as there is with FA or TR, and no truly “optimal” value was found. However, a broad general occlusion can be drawn: there was a general trend of greater accuracy for shorter TEs, which also yield a better signal to noise ratio. The bulk of our work was performed at value of TE of 10 ms.

While systematic and significant variations in the accuracy of the generated signal-concentration curve are noted with TR and FA, there is no obvious *a priori* theoretical reason for such variations based on the SPGR signal equation (5). One obvious question, however, is the relationship between pulse parameters maximizing overall signal in the SPGR equation and the empirically determined optimal pulse parameter sets. Intuitively, it might be assumed that these would be identical, and a signal maximization approach has been used to guide pulse parameter choices by at least one group of investigators [16].

We calculated the signal versus TR and FA manifold, using (5) (Figure 5) and compared the parameters maximizing overall signal to our empirically determined pulse parameter sets. It is found that the optimal combination of TR and FA in terms of accuracy is actually suboptimal set in terms of signal maximization. Whereas for a TR of 40 ms, the accuracy-optimal FA is 30°, the maximal signal strength is obtained at a FA of 15°. Similarly, for a TR of 100 ms, the accuracy-optimal FA is 60°, while signal strength is maximal at 20°.

To further investigate possible theoretical reasons for the systematic variations in accuracy of the predicted signal-concentration curve with TR and FA and to attempt to theoretically derive an optimal set of SPGR pulse parameters, the more general form of the SPGR signal equation was examined (9). When we compared calculated signal curves versus Gd-DTPA concentrations using (5) and (9), for an SPGR sequence TR 40 ms/TE 10 ms/FA of 10°, 30°, 60°, and 90°, we noted no significant differences between the values given by the two equations. As noted, there is excellent agreement between the results predicted by both forms of the signal equation, with only minor deviations being noted at the lowest concentration, and the highest FAs. Hence, use of the more cumbersome equation (9) adds no additional accuracy, and does not allow theoretical derivation of pulse parameter sets.

Finally, we must address the issue of the source of the observed systematic errors in predicted versus observed signal. The methods used in this paper do not answer this question. Most likely, the source of the systematic error errors is twofold: (1) imprecise gradients which cannot achieve the waveforms assumed in the generation of the signal equations and (2) some lack of precision in the signal equations themselves in terms of characterizing steady-state vertical magnetization.

Imprecise gradients imply that the edges of a slice do not experience the same gradients as the central portion of the slice, whereas the SPGR signal equation assumes that every proton within the voxel experiences identical gradient field strength. It has been shown that such slice-shape artifacts vary systematically with FA in gradient echo imaging [28].

A second source of possible systematic error with FA and TR would be some lack of precision in the signal equations, in terms of characterizing steady state vertical magnetization. It is known that with the gradient echo imaging, the spin system reaches a steady-state after the first 20 to 40 excitation pulses, where the loss of longitudinal magnetization by excitation is compensated for by spin lattice relaxation during the TR interval [29]. The theoretical value of this steady state depends on the *T*1, the TR, and the FA [29]. Hence the sort of systematic variations seen between observed and predicted signals as a function of TR and FA would most likely be accounted for by an inaccuracy in the SPGR equations in adequately characterizing the steady state. It is hypothesized that the empirically optimal pulse sequences are those where the steady state is most accurately reflected by the SPGR equations. It is unlikely that other sources of inaccuracy, be they field inhomogeneity, poor shimming, and so forth, could produce these sort of systematic variations observed in our data.

While the etiology of these variations has not been definitively determined, our goal in this paper was to determine the extent of the discrepancy between observed and calculated signals for a sequence that has potential use in the evaluation of quantitative perfusion experiments. Thus, while the exact source of the error is important, our more immediate concern was to establish the validity of using the SPGR sequence on a clinical imaging system.

Our data suggest that the sequence parameters must be optimized if the SPGR sequence is to be used for quantitative mapping of signal to tracer concentrations. As no obvious theoretical way presents itself to choose the optimal pulse parameter sets, it is suggested that a phantom experiment such as that shown in this paper be performed on the clinical imaging system to empirically find the optimal pulse parameter sets which yield the greatest accuracy, and that these pulse parameters then be used in quantitative MR work. 

## Figures and Tables

**Table 1:** Calculated baseline *T1* and *T2* and relaxivities *R1* and *R2*. Values calculated as described in Section 2, and compared to estimates from Su et al. [10]. There is excellent agreement between our results and those of Su et al., suggesting a linear relationship between relaxivity and gadolinium concentration.

*T* (ms)	*R*(ms^−1^ mM^−1^)	R*(ms^−1^ mM^−1^)
*T1*_0_ = 1480	*R*1 = 0.0055	*R*1 = 0.0053
*T2*_0_ = 195	*R*2 = 0.0069	*R*2 = 0.0068

**Table 2:** Absolute average errors (±SD) for TR 40 ms and TR 100 ms as a function of flip angle FA. Absolute average errors vary systematically as a function of FA. For TR 40 ms, the optimal flip angle is 30°, with an absolute average percentage difference PD of 7.9% ± 3%. For a TR of 100 ms, the optimal flip angle is 60°, with an absolute average percentage difference PD of 10.2% ± 4.5%.

TR 40 ms			TR 100 ms		
FA	PD%	PD SD	FA	PD%	PD SD
10°	17.6	4.2	10°	18.3	5.4
30°	7.9	3	30°	16.2	3.7
60°	20.5	7.5	60°	10.2	4.5
90°	42	10.5	90°	28.2	6.7

**Table 3:** Absolute average for a sample sequence for TR 70 ms and TE 10 ms as a function of FA. This data reflects the same systematic variation of accuracy with flip angle, even at fine gradations of the flip angle. Using such fine gradations, it is possible to lower the percentage error to 5%. This occurs at a flip angle of 50°.

TR 70 ms	
FA	PD%
10°	26
20°	23
30°	18
40°	11
50°	5
60°	9
90°	28
